# Treatment of Nocturnal Earache in Children

**Published:** 1883-08

**Authors:** 


### Treatment of Nocturnal Earache in Children.

What physician has not been puzzled to know what to do for the constantly recurring earaches of children at night? Some children cry night after night from pain in one or both ears. They cannot sleep themselves, and will not let others sleep. During the day they are not bothered at all, but as soon as they retire at night the earache begins, and with it the poor mother's trouble begins. All pains are worse at night than in day time. It is quite probable that the ears of such children are more or less painful during the day, but their attention being entirely occupied with their plays, they do not notice the pain. At night, their minds not being otherwise occupied, the slight exacerbation that naturally takes place then is sufficient to keep such children from sleeping.

Now, what is the best treatment for these night earaches in children? The most effectual treatment that I have ever used, or seen recommended for this trouble, is the local use of a solution of sulphate of atropine. I brought this method of treatment to the notice of the profession some years ago, and have had no occasion since to change or even modify it, its effect being so very satisfactory. In fact, I have not yet met with a case of this kind which was not at once relieved by the local use of atropine. The solution is to be simply dropped into the painful ear, and allowed to remain there for ten or fifteen minutes. Then it is made to run out by turning the head over, the ear being wiped off with a dry rag. The solution may be put in cold, though it is better to have it slightly warm, as it does not shock the child so much. From three to five drops should be used at a time.

The strength of the solution must vary according to the age of the child. Under three years, one grain to ounce of water; over three years, two grains to ounce of water; and over ten years, four grains to ounce of water. In a grown person, almost any strength can be used. In a small infant, not more than half a grain to ounce of water should be used. All ages will bear a stronger solution in the ear than in the eye.

The application should be repeated as often as may be necessary. It is not often necessary to use it more than once the same night. Usually, a few applications permanently stop the pain.

The good effect of atropine in painful ears is because of its anodyne power. If physicians will try this plan of treatment in this class of cases, I am sure they will not be disappointed. In acute abscesses of the drum, and acute inflammation of the external meatus, the atropine will only slightly palliate the suffering, but in the recurrent nocturnal earaches of children it is practically a specific.—*Medical Brief.*

